# Robotic versus laparoscopic intracorporeal anastomosis learning curve: a systematic review

**DOI:** 10.1007/s11701-026-03468-5

**Published:** 2026-05-13

**Authors:** Sunny Dhadlie, Kirsten Larkins, Tayla Fay, David Proud, Satish Warrier, Alexander Heriot, Helen Mohan

**Affiliations:** 1 Department of Surgery, Austin Health, Heidelberg, VIC Australia; 2 Department of Surgery, University of Melbourne, Melbourne, VIC Australia; 3 International Medical Robotics Academy (IMRA), Melbourne, VIC Australia; 4 Department of Surgery, Peter MacCallum Cancer Centre, Melbourne, VIC Australia; 5 Department of Surgery, The Alfred Hospital, Melbourne, VIC Australia

**Keywords:** Robotic colorectal surgery, Intracorporeal anastomosis, Learning curve, Surgical training, Robotic surgery, Minimally invasive colorectal surgery

## Abstract

The learning curve for robotic colorectal surgery is increasingly characterised. However, the specific learning curve for intracorporeal anastomosis (IA) remains poorly defined. This review compares robotic and laparoscopic IA with respect to operative time, cases required for proficiency, complication rates, and conversion to extracorporeal anastomosis (EA). A systematic review was conducted in accordance with PRISMA guidelines. The protocol was registered on Open science framework (10.17605/OSF.IO/K4MYZ). On March 18, 2025, OVID and PubMed were searched using the search terms; “laparoscopic,” “robotic,” “anastomosis,” “learning,” “education,” and “training.” Yielding a total of 273 results from PubMed (179) and OVID (94). After screening a total of thirteen studies were included. Thirteen studies met inclusion criteria, including eight comparative analyses and six directly comparing robotic and laparoscopic techniques. Four studies evaluated robotic learning curves without a comparator group, while one was a literature review incorporating institutional experience. Most studies were retrospective (*n* = 8), with two prospective clinical studies and two experimental simulation-based studies. Hybrid approaches (robotic mobilisation with laparoscopic EA) contributed to heterogeneity (Reitz et al. 2018, 10.1007/s00464-018-6074-7). Learning curve assessment was heterogeneous, with most studies using cumulative sum (CUSUM) or risk-adjusted CUSUM (RA-CUSUM) analyses based on operative time or technical performance metrics. Proficiency in robotic IA was reported after approximately 11–29 cases in some series and around 20 cases in others, although larger studies of robotic colorectal surgery demonstrated broader ranges of up to 100 cases. Robotic right hemicolectomy with IA demonstrated a shorter learning curve and reduced anastomosis time compared with laparoscopic IA. Complication rates, conversion rates, and oncological outcomes were comparable between approaches (Gachabayov et al. 2019, Surg Technol Int, 34:163-168). Robotic intracorporeal anastomosis, particularly in right hemicolectomy, appears to have a shorter and less technically demanding learning curve compared with laparoscopic techniques, without compromising safety or oncological outcomes. These findings support a potential role for robotic platforms in facilitating adoption of intracorporeal anastomosis. However, current evidence is limited by retrospective design, small cohorts, and inconsistent definitions of proficiency (Gachabayov et al. 2019, Surg Technol Int, 34:163–168; Van Eetvelde et al. 2022, 10.1007/s11701-022-01514-6).

## Introduction

There has been a notable increase in the use of robotic platforms in colorectal cancer surgery. The current literature is mostly descriptive, featuring single-surgeon or single-centre experiences. These studies share a common aim to show that robotic surgery is non-inferior to laparoscopic approaches in terms of operative time, complications, and oncological outcomes [1–6].

Robotic-assisted surgery may offer advantages for technically demanding tasks such as intracorporeal anastomosis (IA). The enhanced dexterity, articulated instrumentation, and three-dimensional visualisation provided by robotic platforms may facilitate intracorporeal suturing and stapling, improving efficiency and reproducibility during anastomosis formation. While experienced laparoscopic surgeons can achieve high levels of precision using conventional instruments, intracorporeal suturing with straight-stick laparoscopy remains technically challenging and may be associated with greater variability, particularly among less experienced operators. In this context, robotic platforms may make this complex skill more accessible and reduce the technical threshold required to achieve consistent performance. Experimental and early clinical studies have shown that robotic IA is associated with improved technical performance and reduced perceived workload compared with laparoscopic techniques [2–8].

Intracorporeal anastomosis itself has been associated with several benefits compared with extracorporeal anastomosis, including faster recovery of bowel function, reduced postoperative pain, fewer wound-related complications, and shorter hospital stays. As a result, IA is increasingly adopted in minimally invasive right-sided colorectal resections. However, the technical demands of intracorporeal suturing and stapling may represent a barrier to its wider implementation in conventional laparoscopic surgery.

Learning curves in robotic colorectal surgery are commonly evaluated using surrogate performance metrics such as operative time, complication rates, and conversion to open surgery. Analytical methods such as cumulative sum (CUSUM) and risk-adjusted CUSUM (RA-CUSUM) are frequently employed to identify inflection points in performance and estimate the number of cases required to achieve procedural proficiency. However, these methods are applied heterogeneously across studies, with variability in the outcomes assessed and definitions of proficiency, limiting direct comparison between studies [1, 3, 9, 10].

Given the technical complexity of intracorporeal anastomosis, it is plausible that the advantages of robotic platforms—particularly improved dexterity and ergonomics—may have a greater impact on this specific component of colorectal surgery than on the overall procedure. Despite this, most studies have focused on whole-procedure learning curves rather than anastomosis-specific performance. As intracorporeal anastomosis becomes more widely adopted, understanding whether robotic assistance facilitates a shorter or more efficient learning curve for this critical step is of clinical relevance.

While the learning curve for robotic colorectal surgery has been widely studied using metrics such as operative time, oncological quality, complication rates, and conversion to open surgery, the learning curve specifically associated with intracorporeal anastomosis formation remains less clearly defined. Existing studies evaluating robotic IA learning curves have reported varying estimates for the number of cases required to achieve proficiency, often using different analytical methods such as cumulative sum (CUSUM) analysis or sequential case evaluation [1, 3, 9, 10]. Furthermore, direct comparisons between robotic and laparoscopic IA techniques remain limited.

Given the increasing adoption of intracorporeal anastomosis in minimally invasive colorectal surgery, a clearer understanding of the relative learning curves associated with robotic and laparoscopic approaches is important for surgical training, implementation of new techniques, and patient safety.

This systematic review therefore aims to evaluate the available literature comparing learning curves for robotic and laparoscopic intracorporeal anastomosis, with a focus on anastomosis time, number of cases required to achieve procedural proficiency, complication rates, and conversion to extracorporeal anastomosis.

## Methods

### Search strategy

This systematic review was conducted in accordance with the Preferred Reporting Items for Systematic Reviews and Meta-Analyses (PRISMA) guidelines. A comprehensive literature search was performed on 18 March 2025 using the PubMed and Ovid MEDLINE electronic databases. The primary search strategy utilised the following terms: [(laparoscopic) AND (robotic)] AND (anastomosis) AND (learning OR training). This search yielded 179 records from PubMed and 94 records from Ovid MEDLINE. Duplicate records were removed prior to screening. Titles and abstracts were independently reviewed to identify studies evaluating learning curves associated with robotic and laparoscopic intracorporeal anastomosis in colorectal surgery. The search strategy was designed to capture studies examining technical performance, procedural learning curves, and proficiency acquisition related to intracorporeal anastomosis formation using minimally invasive techniques. The study protocol was prospectively registered on the Open Science Framework (10.17605/OSF.IO/K4MYZ).

### Quality assessment

The methodological quality of included clinical studies was assessed using the Newcastle–Ottawa Scale (NOS) for non-randomised studies. This tool evaluates studies across three domains: selection, comparability, and outcome assessment. Studies were independently assessed by two reviewers, with discrepancies resolved by consensus.

Given the nature of included studies, experimental (simulation and animal) studies and studies incorporating institutional experience were not assessed using NOS and were considered separately.

### Study selection and inclusion criteria

All identified records were screened by title and abstract to assess relevance. Abstract screening and full text review for publication selection was completed by two investigators (SD and TF) with a third investigator (HM) resolving conflicts in publication selection. Studies deemed potentially eligible underwent full-text review. The selection process focused on identifying studies that evaluated learning curve parameters associated with robotic and/or laparoscopic intracorporeal anastomosis. Studies not relevant to colorectal surgery, intracorporeal anastomosis, or surgical learning curves were excluded. Following full-text review, 13 studies met the inclusion criteria and were included in the qualitative synthesis. Due to heterogeneity in study design and outcome reporting, meta-analysis was not performed.

#### Inclusion criteria

Studies were included if they met the following criteria:

1. Reported on robotic and/or laparoscopic colorectal surgery involving intracorporeal anastomosis.
2. Evaluated learning curve outcomes, including operative time, anastomosis time, complication rates, conversion rates, or number of cases required to achieve proficiency.
3. Included original clinical data from prospective or retrospective studies; and.
4. Were published in English.

Studies were excluded if they:

1. Evaluated extracorporeal anastomosis exclusively without reporting intracorporeal outcomes.
2. Did not assess learning curve parameters.
3. Were review articles, editorials, conference abstracts without full text, or technical descriptions without clinical outcomes; or.
4. Involved non-colorectal procedures.

### Data extraction

Data were extracted from included studies using a structured approach. Extracted variables included study design, number of patients, surgical platform (robotic or laparoscopic), type of colorectal procedure, surgeon experience, and learning curve metrics. Primary learning curve outcomes included operative time, intracorporeal anastomosis time, number of cases required to achieve proficiency, and conversion rates. Secondary outcomes included postoperative complications and oncological adequacy, where reported. Extracted data were synthesised qualitatively to compare learning curve characteristics between robotic and laparoscopic intracorporeal anastomosis.

## Results

**Fig. 1:**
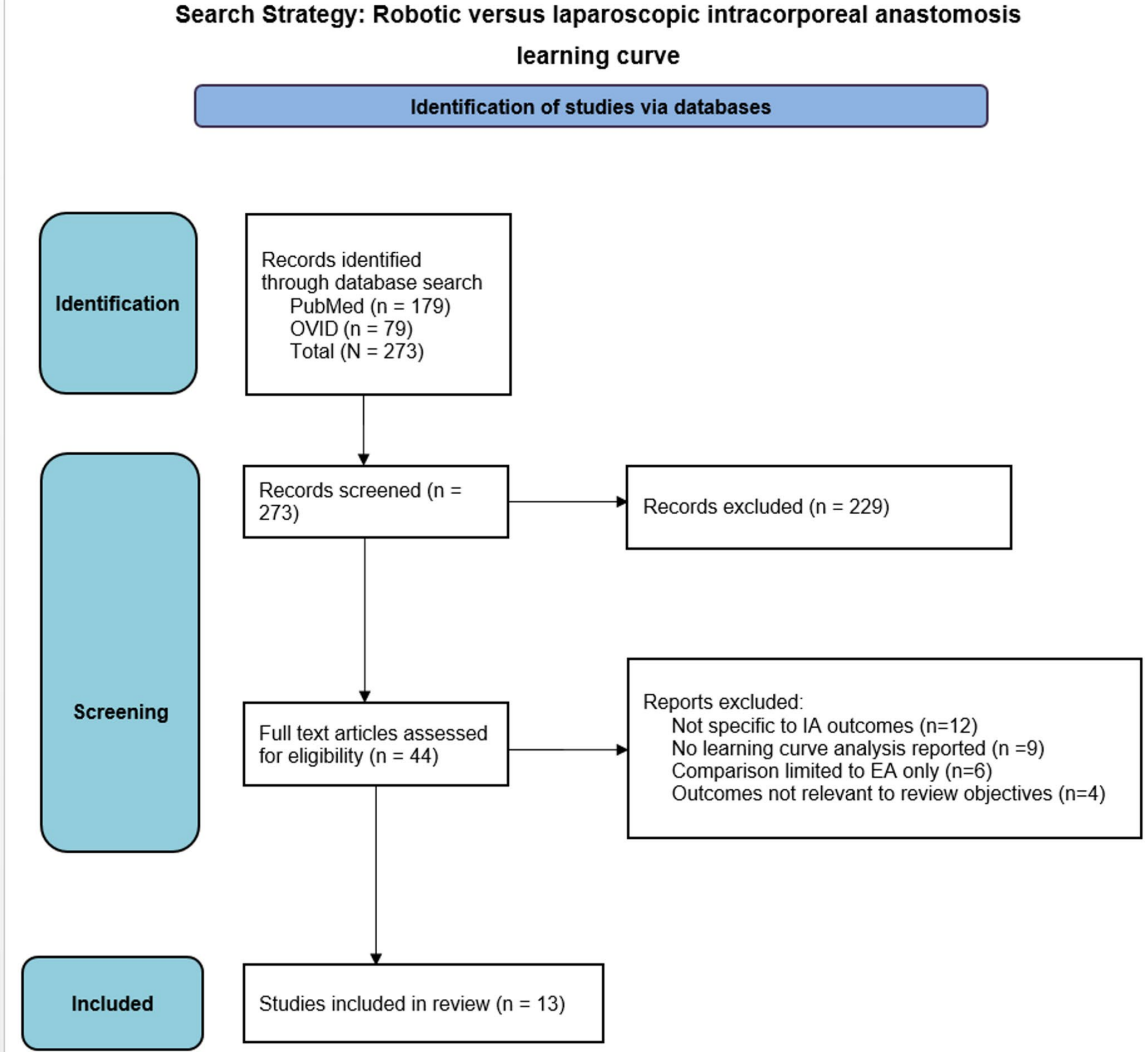
Prisma fl ow diagram of the study selection process

A systematic search of the PubMed and Ovid MEDLINE databases was performed on 18 March 2025 using combinations of the terms *laparoscopic*, *robotic*, *anastomosis*, *learning*, *education*, and *training*. The primary search strategy yielded 179 records from PubMed and 94 from Ovid MEDLINE, resulting in 273 records identified through database searching. After removal of duplicate records, titles and abstracts were screened to identify studies evaluating learning curves associated with robotic and laparoscopic intracorporeal anastomosis in colorectal surgery. Full-text articles were subsequently assessed for eligibility according to the predefined inclusion criteria. A total of 13 studies met the inclusion criteria and were included in the qualitative synthesis. Due to heterogeneity in study design, outcome measures, and reporting of learning curve metrics, a quantitative meta-analysis was not performed (Fig. 1).

### Study characteristics

Thirteen studies evaluating the learning curve of robotic and laparoscopic intracorporeal anastomosis (IA) were included. The majority of studies focused on right hemicolectomy, with comparatively limited data on left-sided colorectal and rectal procedures [1, 3, 9, 10].

Study designs were heterogeneous and included eight comparative studies, four single-arm robotic series, and one study incorporating a literature review with institutional experience [1, 3–6, 9, 11]. In addition, two studies were conducted in experimental settings (porcine model and simulation-based training) [7, 8]. Most clinical studies were retrospective, with a small number of prospective analyses [2, 3]. Considerable variation was observed in surgeon experience, operative technique, and definitions of learning curve endpoints [1, 3, 9, 10].

The methodological quality of included clinical studies was variable. Most studies were retrospective, single-centre cohorts with inherent risks of selection bias and limited control for confounding. Based on Newcastle–Ottawa Scale assessment, the majority of studies were of moderate quality, with common limitations including lack of comparability between groups, absence of blinding, and heterogeneity in outcome assessment.

No randomised controlled trials evaluating clinical outcomes were identified. Experimental and simulation-based studies were not included in the quality assessment (Table 1).

**Table 1:** Summary of characteristics of the included studies

Study	Study design	IA approach	Learning curve metric(s)	Analytical method	Definition of “proficiency”	Process metrics	Clinical outcomes
Reitz et al. [1]	Retrospective, single surgeon	Robotic IA	Operative time, conversion	CUSUM	Plateau in operative time	Operative time	Conversion rate
Gachabayov et al. [2]	Prospective comparative	Robotic vs. Lap IA	Suturing time, knot-tying time	Comparative analysis	Not explicitly defined	Suturing efficiency	Not primary endpoint
Van Eetvelde et al. [3]	Retrospective cohort	Robotic IA	Operative time, complications	CUSUM	Inflection point in operative time	Operative time	Complications, conversion
Hirschburger et al. [4]	Retrospective	Robotic vs. open/lap	Operative time, outcomes	Phase comparison	Not explicitly defined	Operative time	Functional outcomes
Mégevand et al. [5]	Retrospective comparative	Robotic vs. laparoscopic	Operative time, complications	Sequential analysis	Not explicitly defined	Operative time	Complications
Blumberg et al. [6]	Retrospective	Robotic IA	Conversion, operative time	Descriptive	Safe implementation (no conversions)	Operative time	Conversion rate
Haney et al. [7]	Randomised crossover (experimental)	Robotic vs. Lap IA	Technical performance, workload	Comparative scoring	Performance score plateau	Technical performance	Not applicable
Marecik et al. [8]	Simulation study	Robotic vs. Lap IA	Suture quality, task performance	Comparative	Not explicitly defined	Technical skill quality	Not applicable
Parisi et al. [9]	Retrospective cohort	Robotic IA	Operative time, multidimensional metrics	CUSUM/RA-CUSUM	Multiple phase inflection points	Operative time, technical metrics	Complications
Noh et al. [10]	Retrospective	Robotic rectal	Operative time	CUSUM	Plateau in operative time	Operative time	Not IA-specific
Meyer et al. [11]	Prospective cohort	Robotic IA	Operative time, conversion	Sequential	Reduction in operative time	Operative time	Conversion
Formisano et al. [12]	Comparative	Robotic vs. laparoscopic	Operative time, outcomes	Comparative	Not explicitly defined	Operative time	Outcomes
Park et al. [13]	Retrospective	Robotic rectal	Operative time	CUSUM/RA-CUSUM	Multi-phase learning	Operative time	Outcomes

### Clinical studies of robotic and laparoscopic IA

Clinical studies primarily evaluated learning curves using surrogate markers such as operative time, intracorporeal anastomosis time, complication rates, and conversion to extracorporeal anastomosis [1, 3, 5, 9]. Analytical approaches varied and included cumulative sum (CUSUM), risk-adjusted CUSUM (RA-CUSUM), and sequential or phase-based analyses [1, 3, 9, 13].

Across studies, robotic IA demonstrated comparable complication rates, conversion rates, and oncological outcomes to laparoscopic approaches, including during early phases of adoption [3, 5, 6]. Several studies reported reductions in operative time and conversion rates with increasing experience [1, 3, 9]. Reported learning curve thresholds for robotic IA varied widely, ranging from approximately 11 to 44 cases in smaller series [1, 3, 9], with broader ranges reported in larger cohorts depending on the outcome measured [9, 13].

However, definitions of “proficiency” were inconsistent across studies, and outcome measures frequently reflected process metrics rather than comprehensive assessments of surgical competence [13].

### Direct comparative studies of robotic and laparoscopic IA

Only three studies directly compared robotic and laparoscopic intracorporeal anastomosis [2, 7, 8]. These included clinical, experimental, and simulation-based designs. A prospective clinical study reported shorter suturing and knot-tying times with robotic techniques, although overall operative time was longer [2].

Experimental and simulation-based studies demonstrated improved technical performance, enhanced suture quality, and reduced physical and cognitive workload with robotic IA compared with laparoscopic approaches [7, 8].

Despite these findings, the limited number of direct comparisons and heterogeneity in study design preclude definitive conclusions regarding differences in learning curves between platforms [2, 7, 8].

### Experimental and simulation studies

Two studies evaluated intracorporeal anastomosis in non-clinical settings [7, 8]. A randomised crossover study using a porcine model demonstrated improved technical performance and reduced workload with robotic IA [7]. A simulation-based study involving trainees reported improved suture quality and reduced perceived procedural difficulty with robotic techniques, with similar task completion times [8].

These studies provide insight into technical performance and skill acquisition but do not report clinical outcomes.

### Studies incorporating institutional experience

One study incorporated a literature review alongside institutional experience. This study provided descriptive insights into implementation and learning but did not contribute primary comparative clinical data.

#### Learning curve assessment

Learning curve assessment was heterogeneous across studies. Metrics included operative time, anastomosis time, complication rates, and conversion rates, with analytical approaches including CUSUM, RA-CUSUM, and arbitrary phase-based comparisons [1, 3, 9, 13]. Definitions of endpoints such as “efficiency,” “proficiency,” and “safety” varied substantially, limiting comparability between studies [13].

## Discussion

### Principle findings

This systematic review evaluated the learning curve associated with robotic compared with laparoscopic intracorporeal anastomosis (IA) in colorectal surgery. The available evidence suggests that robotic IA is associated with a shorter and less technically demanding learning curve, particularly in right hemicolectomy. This is supported by experimental and clinical studies demonstrating improved technical performance, more efficient suturing, and reduced physical and cognitive workload with robotic platforms compared with laparoscopy [2, 7, 8]. For example, Haney et al. reported superior performance scores and significantly lower NASA-TLX workload measures with robotic IA, while Marecik et al. demonstrated improved suture quality and reduced perceived procedural difficulty among trainees. In a clinical setting, Gachabayov et al. showed significantly shorter suturing and knot-tying times with robotic IA, suggesting more efficient acquisition of intracorporeal suturing skills.

Learning curve thresholds for robotic right hemicolectomy ranged from approximately 11 to 44 cases, with progressive reductions in operative time and conversion rates observed as experience increased [1, 3, 9]. Importantly, robotic IA was performed safely throughout the learning phase, with complication rates and oncological outcomes comparable to laparoscopic approaches [3, 5].

Despite these findings, only three studies directly compared robotic and laparoscopic intracorporeal anastomosis [2, 7, 8]. Most available evidence derives from retrospective series and indirect comparisons, frequently using laparoscopic extracorporeal anastomosis as a comparator [3–5, 12]. This limits the ability to definitively quantify differences in IA-specific learning curves between platforms.

### Mechanisms underlying learning curve differences

Robotic platforms provide technical features such as enhanced dexterity and three-dimensional visualisation that may facilitate intracorporeal tasks.

While robotic platforms offer technical advantages such as enhanced dexterity and visualisation, these features are well established and do not, in isolation, explain differences in learning curves. The extent to which these advantages translate into meaningful improvements in skill acquisition remains uncertain.

Notably, although experimental studies demonstrate improved technical performance and reduced workload with robotic intracorporeal anastomosis [2, 7, 8], clinical outcomes such as complication and conversion rates are largely comparable between robotic and laparoscopic approaches [3, 5, 6]. This suggests that while robotic systems may facilitate certain technical aspects of intracorporeal suturing, their impact on clinically meaningful endpoints is less clear.

Furthermore, the observed differences in learning curves may be influenced by confounding factors, including prior surgical experience and case selection, rather than intrinsic advantages of the robotic platform alone.

### Relationship to broader robotic colorectal surgery learning curves

The findings of this review are consistent with broader literature evaluating robotic colorectal surgery learning curves. Learning curve thresholds ranging from 15 to 45 cases have been reported for robotic right hemicolectomy and robotic low anterior resection [3, 9, 13]. Progressive reductions in operative time and conversion rates observed across studies reflect increasing technical proficiency with robotic platforms [3, 9]. Standardisation efforts, including development of validated video-based competency assessment tools, further support structured evaluation of technical performance during robotic colorectal surgery [15].

Robotic right hemicolectomy is commonly used as an index procedure during robotic adoption, as it allows surgeons to develop robotic skills in a less anatomically constrained environment [6, 11]. Intracorporeal anastomosis represents a key technical component of this procedure. In contrast, intracorporeal anastomosis is already routinely performed during laparoscopic left-sided colorectal surgery, limiting the ability to isolate IA-specific learning curves in this setting [10, 13].

Intracorporeal anastomosis itself offers clinical advantages compared with extracorporeal techniques, including reduced bowel manipulation and improved recovery [5, 12]. Meta-analyses have further demonstrated comparable or improved short-term outcomes with intracorporeal techniques during robotic right colectomy [16, 17]. Robotic platforms may therefore facilitate wider adoption of intracorporeal techniques by reducing technical barriers associated with laparoscopic suturing.

### Clinical and training implications

The shorter learning curve associated with robotic IA has important implications for surgical training and clinical practice. Several studies demonstrated safe implementation of robotic IA during early adoption without increased complication rates [3, 5, 6]. Reduced technical difficulty and improved ergonomics may facilitate safer adoption of minimally invasive colorectal surgery.

Robotic platforms may also enhance surgical training by allowing surgeons to acquire intracorporeal suturing skills more efficiently. Intracorporeal anastomosis represents a key procedural component that incorporates a broad range of fundamental robotic skills, including endowrist manipulation, camera control and clutching, depth perception, tissue handling, energy application, stapling, and suturing. As such, IA may serve as a valuable index task for developing proficiency across the robotic skill set. Structured training pathways incorporating robotic simulation, objective performance assessment, and stepwise progression may further optimise skill acquisition [14, 15]. Adjuncts such as simulation and three-dimensional modelling have also been explored to support training in minimally invasive colorectal procedures [18].

Robotic right hemicolectomy with intracorporeal anastomosis therefore appears to be an appropriate index procedure for surgeons transitioning to robotic colorectal surgery, allowing development of core robotic technical skills prior to more complex pelvic procedures [6, 11].

### Limitations of the evidence

The overall quality of included studies was moderate. Only three studies directly compared robotic and laparoscopic intracorporeal anastomosis [2, 7, 8], and most included studies were retrospective single-centre series [1, 3–6, 9, 11]. Many comparative studies used laparoscopic extracorporeal anastomosis rather than laparoscopic IA as a comparator, limiting direct evaluation of IA-specific learning curves [3–5, 12]. The absence of randomised controlled trials and heterogeneity in outcome reporting further limit the strength of conclusions.

Substantial heterogeneity existed in study design, surgeon experience, operative technique, and definitions of proficiency. Operative time was the most used surrogate marker of proficiency, although it does not fully capture surgical competence [9, 13]. More comprehensive assessment incorporating complication rates, oncological outcomes, and objective technical performance metrics is needed. Recent studies have highlighted the value of multidimensional learning curve assessment in evaluating intracorporeal anastomosis performance [19].

A wide range of assessment tools have been proposed to evaluate technical performance, including global rating scales such as GEARS and GOALS, objective performance metrics (OPMs) derived from instrument motion analysis and increasingly supported by artificial intelligence, as well as multimodal proficiency-based frameworks incorporating technical, clinical, and procedural outcomes. Consensus guidance has also emphasised the importance of structured and standardised approaches to robotic right hemicolectomy and intracorporeal anastomosis [20]. However, there remains no clear agreement on which metrics are most relevant for defining proficiency or predicting clinically meaningful outcomes. This variability in outcome measurement further limits comparison across studies and highlights the need for standardised, validated assessment frameworks.

The current evidence base is its predominance of right-sided colorectal procedures. Most included studies evaluated intracorporeal anastomosis in the context of right hemicolectomy, which is commonly used as an index procedure during adoption of minimally invasive and robotic colorectal surgery. In contrast, data on left-sided and rectal procedures are limited and often confounded by differences in operative technique, anatomical constraints, and routine use of intracorporeal anastomosis across both laparoscopic and robotic approaches. As such, the generalisability of these findings to left-sided colorectal and rectal surgery remains uncertain.

A key limitation in interpreting learning curves for robotic intracorporeal anastomosis is the confounding effect of prior surgical experience. Many included studies involved surgeons with established laparoscopic expertise or early adopters of robotic platforms, which may shorten observed learning curves and influence technical performance metrics. As a result, it is difficult to determine whether reported improvements reflect true acquisition of novel robotic skills or transfer of existing laparoscopic competencies [6, 11].

The extent to which intracorporeal anastomosis represents a distinct technical paradigm versus an extension of laparoscopic skill remains unclear. While some studies suggest that robotic platforms facilitate more efficient acquisition of intracorporeal suturing skills, these findings are often derived from cohorts with prior minimally invasive experience. Importantly, few studies evaluate learning curves in surgeons without prior laparoscopic expertise, limiting the ability to define a baseline robotic learning trajectory.

Future research should aim to stratify outcomes by prior surgical experience and include novice operators to better distinguish between skill transfer and true robotic learning.

### Future directions

Standardised definitions of learning curve phases and multidimensional performance metrics should be used to more accurately characterise skill acquisition in intracorporeal anastomosis. Future studies should prioritise IA-specific outcomes, including anastomosis time, technical quality, and complication rates, rather than relying solely on whole-procedure metrics such as operative time. Evaluation of surgeons with varying levels of experience is required to better define the true learning curve associated with robotic IA, particularly among novice operators. Additionally, studies should examine intracorporeal anastomosis across different colorectal procedures to determine the generalisability of learning curve findings beyond right hemicolectomy.

## Conclusion

Robotic intracorporeal anastomosis suggests potential for a shorter and less technically demanding learning curve compared with laparoscopic intracorporeal anastomosis, particularly in right hemicolectomy [1, 3, 9]. Robotic platforms facilitate improved technical performance and reduced cognitive and physical workload [2, 7, 8], without compromising safety or oncological outcomes [3, 5]. However, these findings are predominantly derived from right-sided procedures, and further studies are required to determine their applicability to left-sided and rectal colorectal surgery. Interpretation is limited by heterogeneity in learning curve definitions, limited direct comparative data, and confounding from prior surgical experience. The predominance of right-sided procedures further restricts generalisability. Standardised definitions of proficiency and prospective studies across varying levels of surgeon experience and colorectal procedures are required [2, 7, 8]. Intracorporeal anastomosis represents a key procedural component for acquisition of core robotic skills and may support structured training pathways [14, 15].

## Data Availability

All data generated or analysed during this study are included in this published article and its supplementary information files.
